# Robot Imitation Learning of Social Gestures with Self-Collision Avoidance Using a 3D Sensor

**DOI:** 10.3390/s18072355

**Published:** 2018-07-20

**Authors:** Tan Zhang, Wing-Yue Louie, Goldie Nejat, Beno Benhabib

**Affiliations:** 1 Autonomous Systems and Biomechatronics Lab, Department of Mechanical and Industrial Engineering, University of Toronto, Toronto, ON M5S3G8, Canada; geoffrey.louie@utoronto.ca (W.-Y.L.); nejat@mie.utoronto.ca (G.N.); 2 College of Computer Science and Software Engineering, Shenzhen University, Shenzhen 518060, China; 3 Intelligent Robotics Lab, Department of Electrical and Computer Engineering, Oakland University, Rochester, MI 48309, USA; 4 Computer Integrated Manufacturing Lab, Department of Mechanical and Industrial Engineering, University of Toronto, Toronto, ON M5S3G8, Canada; benhabib@mie.utoronto.ca

**Keywords:** imitation learning, social robots, multi-jointed arm gestures, self-collision avoidance

## Abstract

To effectively interact with people, social robots need to perceive human behaviors and in turn display their own behaviors using social communication modes such as gestures. The modeling of gestures can be difficult due to the high dimensionality of the robot configuration space. Imitation learning can be used to teach a robot to implement multi-jointed arm gestures by directly observing a human teacher’s arm movements (for example, using a non-contact 3D sensor) and then mapping these movements onto the robot arms. In this paper, we present a novel imitation learning system with robot self-collision awareness and avoidance. The proposed method uses a kinematical approach with bounding volumes to detect and avoid collisions with the robot itself while performing gesticulations. We conducted experiments with a dual arm social robot and a 3D sensor to determine the effectiveness of our imitation system in being able to mimic gestures while avoiding self-collisions.

## 1. Introduction

Social robots are currently being developed to interact with humans by perceiving human behavior [1] and displaying natural human-like gestures such as waving to greet humans or pointing to objects to guide their visual focus of attention [2,3,4]. Trajectories to generate these human-like gestures are often programmed and implemented by expert roboticists. Imitation learning provides a natural mode of human-robot interaction for non-experts to teach a robot arm gestures by having a robot observe, using visual sensors, the teacher’s physical demonstrations of the gestures and then map these observed motions onto the robot’s own embodiment [5,6,7].

### 1.1. Related Work

#### 1.1.1. Imitation Learning

A common approach for imitation learning is to sense the 3D arm joint positions of a demonstrator and solve for the inverse kinematics problem to determine the joint angles between each of the demonstrator’s arm segments [8]. The demonstrator joint angles can then be directly mapped to the robot’s arms for robots with the same degrees of freedom (DOF) of a human (e.g., the Nao robot). The benefit of utilizing an inverse kinematics model for trajectory representation is that all arm segments are represented at once. These models can be solved using an analytical approach [9], iterative Jacobian [10], or neuro-fuzzy system methods [10]. It was found in [10] that analytical approaches and iterative Jacobian methods require more complex models that cannot be solved in real-time, while neuro-fuzzy system methods require large amounts of training data to generate accurate models. The geometrical approach in [11] utilizes triangles to model the joint angles for human arm motions, which can be used for directly mapping human arm motions to a robot. The geometric-based imitation learning system presented in this paper is inspired by the triangle-based approach presented in [11].

#### 1.1.2. Imitation Learning with Self-Collision Avoidance

Imitation learning alone is not sufficient to mimic human arm gestures on a robot as differences in the embodiment can lead to self-collisions (which can cause damage to the robot) and the inability to implement certain movements. Hence, self-collision avoidance techniques are necessary to generate collision-free trajectories that are close approximations of a demonstrator’s arm gestures. Presently, there is some work that detects if self-collisions will occur given a motion trajectory [12,13]; however, they are not capable of generating alternative trajectories to avoid these self-collisions. These self-collision approaches model a robot’s body, arms and/or head using bounding volumes, such as spheres and circular capsules (i.e., cylinders capped with spheres) and calculate the distance between the bounding volumes. Collisions are defined as scenarios where the distance between two bounding volumes is less than the sum of their two radii. The work in [12] proposed a collision detection method for a vine pruning robot by modeling plant canes and the robot arm as capsules to check for collisions between the robot arm and the vine. This approach only considers the collisions between two capped ends and the coincidence between two capsules, without taking into account the collisions between two cylinders, whose spatial relations are parallel, perpendicular, or skew. Similarly, in [13] the robot’s arms are modeled as circular capsules while elliptical capsules (i.e., elliptical cylinders capped by ellipsoids) were used to fit the humanoid torso shape. Namely, spatial relations between capsules were determined by modeling each capsule as a straight line of infinite length and verifying if the lines intersect. However, such an approach can lead to incorrect collision detections as the capsules have finite length.

More recently, some preliminary work has been done to integrate self-collision avoidance techniques into robot imitation learning systems [14,15,16,17,18]. In [14], continuous null-space projections were used to avoid the potential collisions between two Kuka LWR robotic arms being controlled through an imitation learning system using a Kinect sensor. Namely, self-collision avoidance was accomplished by constraining arm joint movements within the null-space projection of the collision-free arm configuration. However, the approach was limited to self-collision avoidance between only two arms and did not account for self-collision avoidance with multiple body parts (e.g., head, arms, and torso). In [15], an imitation learning system with self-collision avoidance utilized six optimal cameras and 38 markers attached to a demonstrator to sense his/her joint positions during a movement. An inverse kinematics-based approach was used to determine demonstrator joint angles from the sensed joint positions and a collision-free motion trajectory was mapped to the robot by solving for a trajectory that meets the robot’s joint angle and self-collision constraints. Namely, self-collisions were detected by determining the distance between any two 3D points on the robot. However, such a self-collision detection approach is computationally expensive and would not work in real-time as it requires a pairwise distance calculation between all 3D points on a robot. In [16], a Kinect sensor-based imitation learning system with self-collision avoidance was developed for a robot with dual arms mounted on a torso. The sensed 3D joint positions of the demonstrator were mapped directly onto a robot by inputting the positions into a cartesian impedance controller. Self-collision avoidance for this imitation learning system was accomplished by modeling the robot’s arms as two-line segments and the torso as a rectangular plane. Namely, the minimum distance between the two-line segments and all the distances from the line segments to the plane were continuously monitored to detect collisions between the robot’s body parts (i.e., arms and torso). Detected collisions were then used to modify the robot motion trajectories by moving the body parts in the opposite direction of the contact at a speed proportional to the distance of the body parts. However, such a technique has the potential to reduce the similarity of the robot poses to the demonstrator and can result in jerky motions due to rapid changes in speed when avoiding collisions. In [17], a Kinect sensor-based imitation learning system with self-collision avoidance was developed for the humanoid robot Nao. Namely, an inverse-kinematics-based approach was applied to determine demonstrator joint angles from sensed joint positions and directly map them to the Nao robot’s joints. A self-collision avoidance technique was also presented that models the robot links as cylinders with spherical ends. The criterion for a collision was defined as situations where the shortest distance between two-line segments was less than the sum of the cylinder radii. When a collision was detected, the robot just stopped moving. However, for such a collision avoidance technique, the demonstrator’s poses need to be sampled at a fast-enough rate in order to avoid the motion between two collision free poses introducing a collision. In [18], a similar Kinect sensor-based imitation learning system with self-collision was developed for the humanoid robot Nao. Namely, the robot’s workspace was first empirically modeled off-line by manually sampling robot joint poses to identify if a collision would occur. The workspace model was then utilized online to solve for collision-free joint poses to imitate a demonstrator’s poses by determining if a solution was within the collision-free workspace of the robot. However, the accuracy was limited to the number of samples used to model the workspace and could not account for all possible collision/collision-free poses.

To date, imitation learning systems with self-collision avoidance are limited as they are: (1) capable of modelling only collisions between two body parts [14]; (2) computationally expensive and therefore cannot be used in real-time [15]; (3) unable to account for all collisions/collision-free poses during a demonstration [17,18]; and (4) unsuitable for gesture demonstrations due to rapid joint speed changes as a result of the collision avoidance technique [16]. Therefore, in this paper, we propose a real-time gesture imitation learning system that can address these limitations by detecting all self-collisions for a robot with multiple body parts. The novelty of this approach is that it optimizes a collision-free motion trajectory while minimizing the distance from the demonstrator’s trajectory. The system utilizes a geometric-based approach to map human joint angles to robot joint angles and a kinematical-based approach for self-collision avoidance.

## 2. Gesture Imitation Learning System

**Figure 1:**
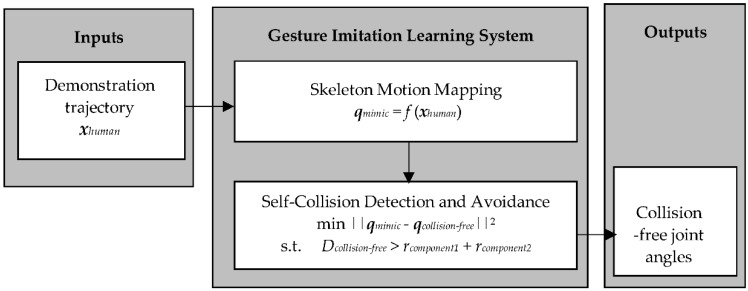
Diagram of the gesture imitation learning system.

The proposed gesture imitation learning system is presented in Figure 1. The gesture imitation leaning system has two main modules: (1) a skeleton motion mapping module; and (2) a self-collision detection and avoidance module. Namely, the skeleton motion mapping module takes as input a sequence of demonstrator poses (i.e., joint positions in 3D space), herein referred to as a demonstration trajectory, and solves for the sequence of demonstrator joint angles executed to attain this sequence of poses. The self-collision detection and avoidance module then detects for potential self-collisions when the sequence of joint angles is directly mapped to the robot. If a collision is detected for a robot joint angle configuration in the sequence it solves for a collision-free joint angle configuration that closely imitates the human demonstrator. Finally, the sequence of collision-free joint angle configurations is outputted to be executed by the robot.

### 2.1. Skeleton Motion Mapping Module 

**Figure 2:**
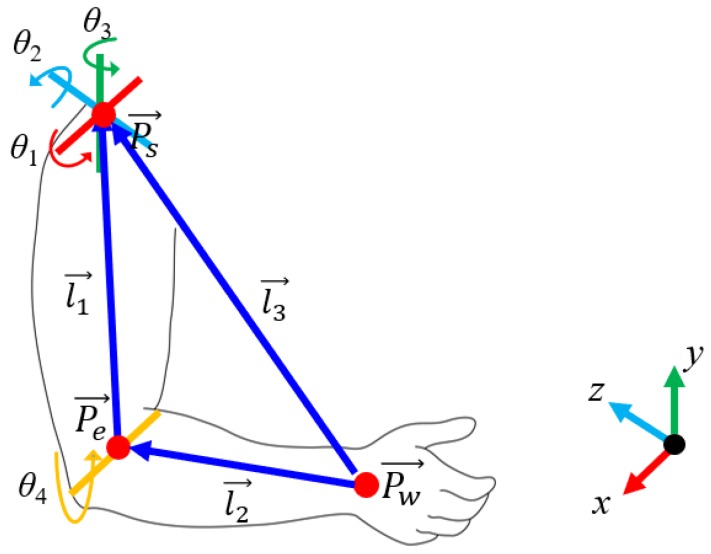
The human arm model.

The skeleton motion mapping module first obtains the demonstration trajectory and applies a geometric-based approach to each of the demonstrator’s poses, ***x****_human_*, to obtain a mapping to the robot’s pose, ***q****_mimic_*. As shown in Figure 2, the demonstrator’s arm is modeled with four degrees of freedoms (DOFs). There are three DOFs in the shoulder and one DOF in the elbow. We first determine the shoulder roll (*θ*_1_), pitch (*θ*_2_), and yaw (*θ*_3_) angles using the following equations:

(1) $$\;\theta_{1}=arcsin\left(\frac{\underset{l1y}{\to }}{\sqrt{{\underset{l1x}{\to }}^{2}+{\underset{l1y}{\to }}^{2}+{\underset{l1z}{\to }}^{2}}}\right)\;$$

(2) $$\;\theta_{2}=arcsin\left(\frac{\underset{d1x}{\to }}{\sqrt{{\underset{l1x}{\to }}^{2}+{\underset{l1y}{\to }}^{2}+{\underset{l1z}{\to }}^{2}}}\right)\;$$

(3) $$\;\theta_{3}=arcsin\left(\frac{\underset{d2x}{\to }}{\sqrt{{\underset{l2x}{\to }}^{2}+{\underset{l2y}{\to }}^{2}+{\underset{l2z}{\to }}^{2}}}\right)\;$$

where $\vec{l_{1x}}$, $\vec{l_{1y}}$, and $\vec{l_{1z}}$ are the projections of *l*_1_ on the axes of *x*, *y* and *z*, respectively. Similarly, $\vec{l_{2x}}$, $\vec{l_{2y}}$, and $\vec{l_{2z}}$ are the projections of *l*_2_ on the axes of *x*, *y* and *z*, respectively. $\vec{l_{1}}$, $\vec{l_{2}}$, and $\vec{l_{3}}$ are obtained as follows:

(4) $$\;\vec{l_{1}}=\vec{P_{e}}-\vec{P_{s}}\;$$

(5) $$\;\vec{l_{2}}=\vec{P_{w}}-\vec{P_{e}}\;$$

(6) $$\;\vec{l_{3}}=\vec{P_{w}}-\vec{P_{e}}\;$$

where $\vec{P_{w}}$, $\vec{P_{e}}$, and $\vec{P_{s}}\;$ are the positions of the wrist, elbow and shoulder, and ***x****_human_ =* {$\vec{P_{w}}$, $\vec{P_{e}}$, $\vec{P_{s}}$}.

We then determine the elbow roll angle, *θ*_4_, with the following equation:

(7) $$\;\theta_{4}=\arccos\left(\frac{{\left|l_{3}\right|}^{2}-\left|l_{1}{|}^{2}-\right|l_{2}{|}^{2}}{2\left|l_{1}\right|\left|l_{2}\right|}\right)\;$$

where $\left|\vec{l_{1}}\right|$ and $\left|\vec{l_{2}}\right|$ are the upper arm and forearm of the left/right arm, respectively. $\left|\vec{l_{3}}\right|\;$ is the length from the shoulder to the elbow, as shown in Figure 2.

The shoulder and elbow angles of the demonstrator are then mapped directly to robot’s pose, ***q****_mimic_*, since we are focusing on imitation learning for robots with a humanoid upper torso. Namely, the robot’s pose can be defined as the set ***q****_mimic_ =* {$\theta_{1},\theta_{2},\cdots ,\theta_{u}\}$, and *u* is the number of the robot joints. The robot’s pose is then input to the self-collision detection and avoidance module to avoid self-collisions due to differences in embodiment between the robot and human demonstrator.

### 2.2. Self-Collision Detection and Avoidance Module

The self-collision detection and avoidance module is represented by two submodules: (1) the self-collision detection submodule that utilizes a bounding-volume-based approach to detect collisions between any two body parts for a robot pose; and (2) the collision avoidance submodule that solves for the closest collision-free joint angles that mimic the demonstrator’s poses. 

To avoid the possible collision points, the module first checks the distance between any two components. Using a bounding-volume-based approach, the components are modeled as spheres or capsules with a radius *r*. Our approach obtains the position of each joint based on ***q****_mimic_* by forward kinematics, and the distance *D* between two components whose radii are *r*_1_ and *r*_2_. If *D* > *r*_1_ *+ r*_2_, there is no collision between the two components. If there is a collision between two components, the module finds a set of new angles ***q****_collision-free_* that has a minimum difference with ***q****_mimic_*, to ensure that there is a minimum distance *D* between the two volumes.

minimize   ||***q***_*mimic*_ − ***q***_*collision-free*_||^2^ subject to      *D* > *r*_1_ + *r*_2_ (8)

Finally, ***q****_collision-free_* is sent to the robot to implement.

#### 2.2.1. Self-Collision Detection

The self-collision detection submodule is used to determine whether two body parts of the robot will be in collision given a robot pose ***q****_mimic_*. Herein, we model a robot’s body parts as either capsules (e.g., arms, torso) or spheres (e.g., head). A sphere is defined using its centroid and radius. A capsule is a cylinder with two half spheres at its two ends, which is defined by the positions of its two end points and the radius of the cylinder. We define a robot pose to have no self-collisions when the distance between two body parts is larger than the summation of the radii of the two body part models:

*D_kj_* − (*r_k_* + *r_j_*) > 0, *k* = 1, …, *n*; *j* = 1, …, *n* (9)

where *D_kj_* is the shortest distance between two modeled robot body parts, body parts *k* and *j*. *r_k_* and *r_j_* are the radii of robot body parts *k* and *j*, respectively. *n* is the total number of robot body parts.

##### Collision Checks between a Sphere and a Capsule

**Figure 3:**
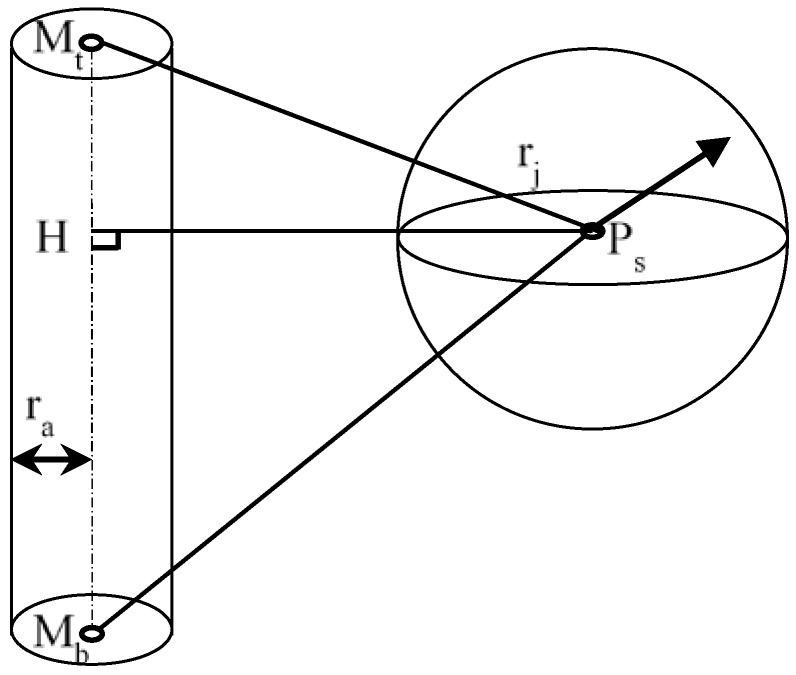
Collison check between a sphere and a capsule.

The collision checks of a sphere and a capsule are represented by the distance between a point and a line segment in 3D space. Let *P_s_*, *M_t_*, and *M_b_* represent the center of sphere *S*, and the two end points of capsule *M*, respectively, as shown in Figure 3. $\vec{M_{t}M_{b}}$ is the axis of capsule *M*. *H* is the foot of the common normal of point *P_s_* on $\vec{M_{t}M_{b}}$.

The following different cases are considered for calculating the distance between a sphere and a capsule:

(1) If $\vec{P_{s}M_{t}}\cdot \vec{M_{t}M_{b}}\;\leq \;0$, the intersection of the perpendicular line lies on the extended line of $\vec{M_{t}M_{b}}$ near Point *M_t_*, and the shortest distance is $\left|\vec{P_{s}M_{t}}\right|$.
(2) If $\vec{P_{s}M_{t}}\cdot \vec{M_{t}M_{b}}\;\geq \;\left|\vec{M_{t}M_{b}}\right|$, the intersection of the perpendicular line lies on the extended line of $\vec{M_{t}M_{b}}$ near Point *M_b_*, and the shortest distance is $\left|\vec{P_{s}M_{b}}\right|$.
(3) If $0\leq \vec{P_{s}M_{t}}\cdot \vec{M_{t}M_{b}}\;\leq \;\left|\vec{M_{t}M_{b}}\right|$, the intersection of the perpendicular line lies on $\vec{M_{t}M_{b}}$, and the shortest distance is $\left|\vec{P_{s}H}\right|$.

##### Collision Checks between Two Capsules

The collision checks of two capsules become the distance between two line-segments in 3D space. Let *M_t_*, *M_b_*, *N_t_* and *N_b_* represent the two end points of capsule *M* and capsule *N*, respectively. $\vec{M_{t}M_{b}}$ is the axis of *M* and $\vec{N_{t}N_{b}}$ is the axis of *N.*

**Figure 4:**
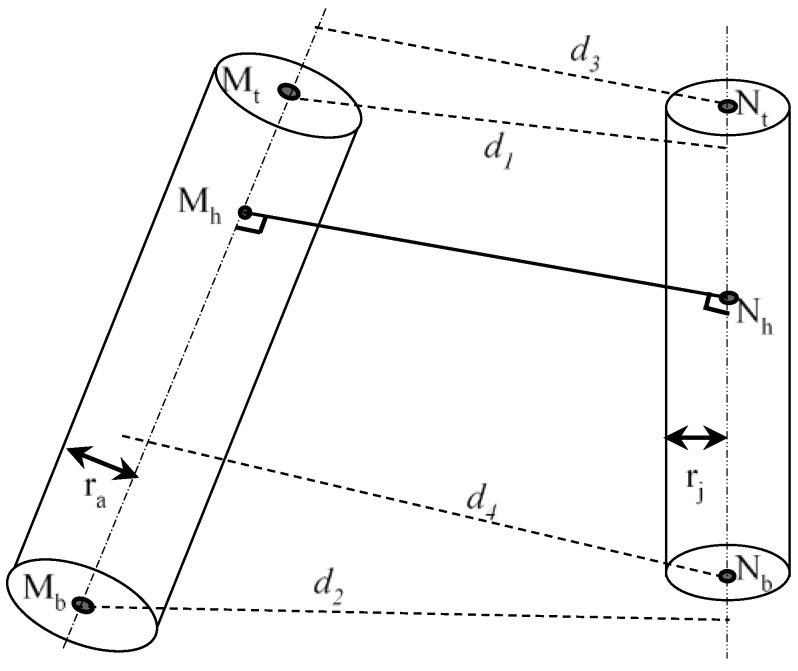
Collison checks between two capsules (two axes are skew).

**Figure 5:**
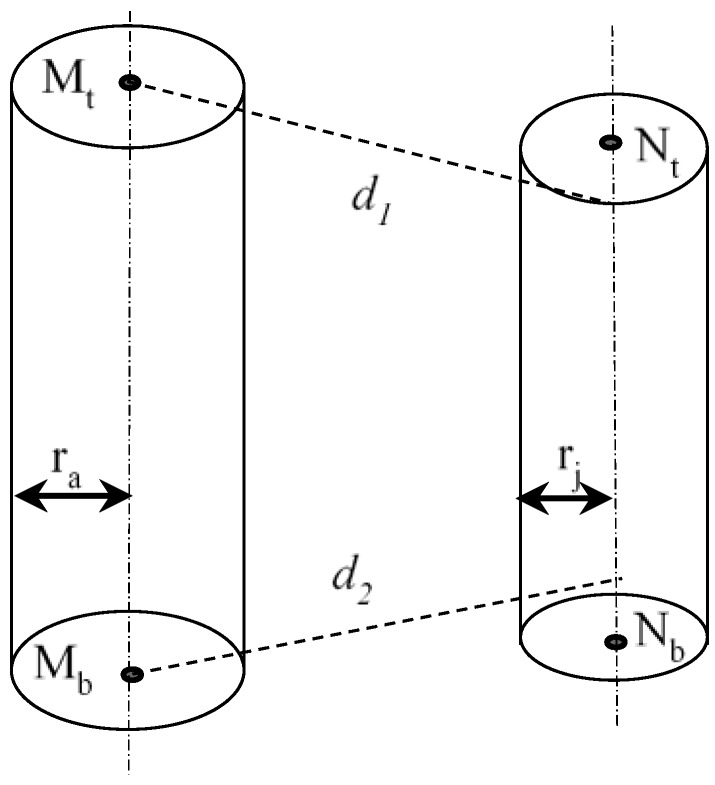
Collison checks between two capsules (two axes are parallel).

**Figure 6:**
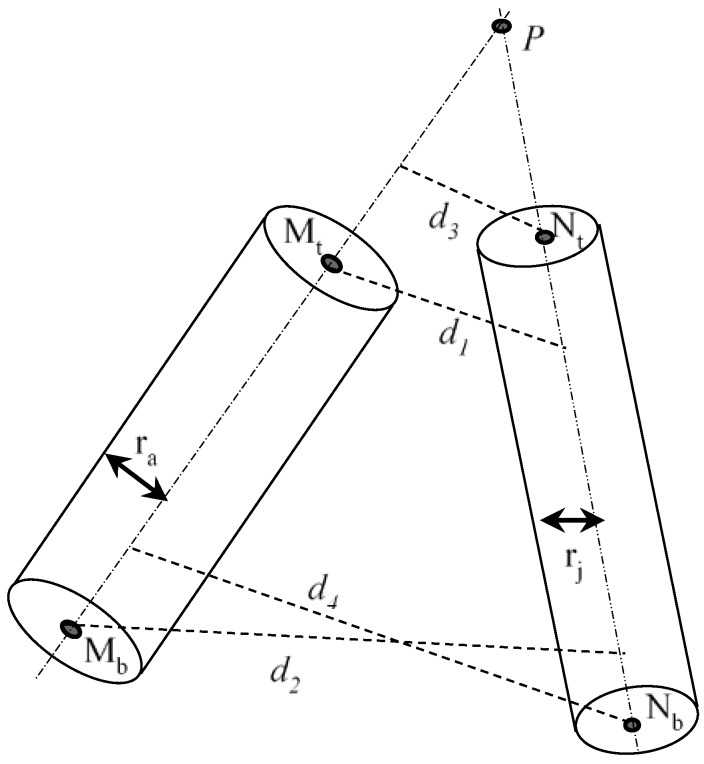
Collison checks between two capsules (two axes are intersecting).

The following are several cases for calculating the distance between two capsules:

(1) If $\vec{M_{t}M_{b}}\cdot \vec{M_{t}N_{t}}\cdot \vec{M_{t}N_{b}}\;\neq \;0$, the two lines $\vec{M_{t}M_{b}}$ and $\vec{N_{t}N_{b}}$ are skew lines, as shown in Figure 4. There are two situations as follows:
  (i) If both the intersections *M_h_* and *N_h_* lie on the two segments, i.e., $\left|\vec{M_{t}M_{h}}\right|+\left|\vec{M_{h}M_{b}}\right|=\left|\vec{M_{t}M_{b}}\right|$ and $\left|\vec{N_{t}N_{h}}\right|+\left|\vec{N_{h}N_{b}}\right|=\left|\vec{N_{t}N_{b}}\right|$, then the shortest distance is the perpendicular distance $\left|\vec{M_{h}N_{h}}\right|$.
  (ii) If one of the two intersections, *M_h_* or *N_h_*, do not lie on the segment, i.e., $\left|\vec{M_{t}M_{h}}\right|+\left|\vec{M_{h}M_{b}}\right|\neq \left|\vec{M_{t}M_{b}}\right|$ or $\left|\vec{N_{t}N_{h}}\right|+\left|\vec{N_{h}N_{b}}\right|\neq \left|\vec{N_{t}N_{b}}\right|$, the shortest distance is the smallest among $\left|\vec{M_{h}N_{h}}\right|$, and *d*_1_, *d*_2_, *d*_3_ and *d*_4_, as shown in Figure 4. *d*_1_, *d*_2_, *d*_3_ and *d*_4_ are the perpendicular distance from point *M_t_* to $\vec{N_{t}N_{b}}$, the perpendicular distance from point *M_b_* to $\vec{N_{t}N_{b}}$, the perpendicular distance from point *N_t_* to$\;\vec{M_{t}M_{b}}$, and the perpendicular distance from point *N_b_* to $\vec{M_{t}M_{b}}$, respectively.
(2) If the two lines $\vec{M_{t}M_{b}}$ and $\vec{N_{t}N_{b}}$ are proportional, i.e., $\vec{M_{t}M_{b}}$ = $k\cdot \vec{N_{t}N_{b}}$, where k ≠ 0, the two lines are parallel, as shown in Figure 5. The shortest distance will be the smaller of *d*_1_ and *d*_2_.
(3) If the two lines $\vec{M_{t}M_{b}}$ and $\vec{N_{t}N_{b}}\;$are neither skew nor parallel, the two lines are intersected at *P*, as shown in Figure 6. There are two situations as follows:
  (i) If *P* lies on the two segments, i.e., $\left|\vec{PM_{t}}\right|+\left|\vec{M_{t}M_{b}}\right|=\left|\vec{PM_{b}}\right|$ and $\left|\vec{PN_{t}}\right|+\left|\vec{N_{t}N_{b}}\right|=\left|\vec{PN_{b}}\right|$, the shortest distance is zero. Let the two lines $\vec{M_{t}M_{b}}$ and $\vec{N_{t}N_{b}}$ be represented as vectors $\vec{m}\left(t\right)=\vec{M_{t}}+\vec{M_{t}M_{b}}\;\cdot t$ and $\vec{n}\left(t\right)=\vec{N_{t}}+\vec{N_{t}N_{b}}\;\cdot s$, the intersection of the two segments, i.e., the position of *P* is the solution of $\vec{m}\left(t\right)=\vec{n}\left(t\right)$.
  (ii) If *P* does not lie on one of the two segments, i.e., $\left|\vec{PM_{t}}\right|+\left|\vec{M_{t}M_{b}}\right|\neq \left|\vec{PM_{b}}\right|$ or $\left|\vec{PN_{t}}\right|+\left|\vec{N_{t}N_{b}}\right|\neq \left|\vec{PN_{b}}\right|$, the shortest distance is the smallest among *d*_1_, *d*_2_, *d*_3_ and *d*_4_.

#### 2.2.2. Self-Collision Avoidance

Given a self-collision has been detected, the self-collision avoidance submodule determines robot joint angles that best mimic a human demonstrator’s pose. Namely, we define self-collision avoidance as an optimization problem which solves for the set of robot joint angles which are collision-free, ***q****_collision-free_*, and has the minimum joint angle difference with a demonstrated pose ***q****_mimic_*. The objective function, *e*, for the optimization is defined as:

(10) $$\mathrm{minimize}\;e=\sum_{i=1}^{u}{\left(\theta_{i}-\theta_{i}^{\prime }\right)}^{2}\;\mathrm{subject}\;\mathrm{to}\;D_{kj}\;-\;\left(r_{k}\;+\;r_{j}\right)\;>\;0,\;k\;=\;1,\;\ldots ,\;n;\;j\;=\;1,\;\ldots ,\;n$$

where *i* is the robot joint angle being optimized, *u* is the total number of robot joints, *θ_i_* and $\theta_{i}^{\prime }\;$are, respectively, the demonstrated joint angle and collision-free joint angle of joint *i*. The constraint for the optimization is that all the robot body parts (i.e., head, arms, upper torso, and lower torso) must be collision free. *D_kj_* is the shortest distance between two modeled robot body parts, body parts *k* and *j*. *r_k_* and *r_j_* are the radii of robot body parts *k* and *j*, respectively. *n* is the total number of robot body parts. 

**Figure 7:**
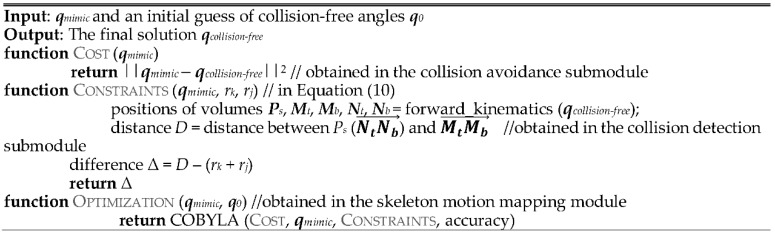
Pseudo-code for searching collision-free angles.

In this work, we used a constrained optimization by linear approximation (COBYLA) algorithm [19] to solve the optimization problem. COBYLA is a derivation-free optimization method for constrained problems [20]. The specific algorithms we have designed for this optimization are presented in Figure 7.

## 3. Imitation Learning with the Tangy Robot

**Figure 8:**
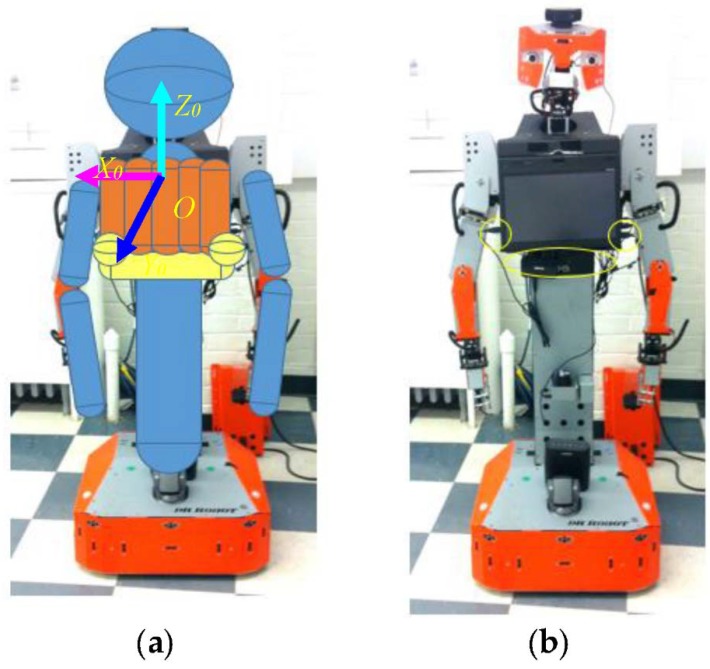
(**a**) The Tangy robot; (**b**) modeled with three spheres and eleven capsules.

**Figure 9:**
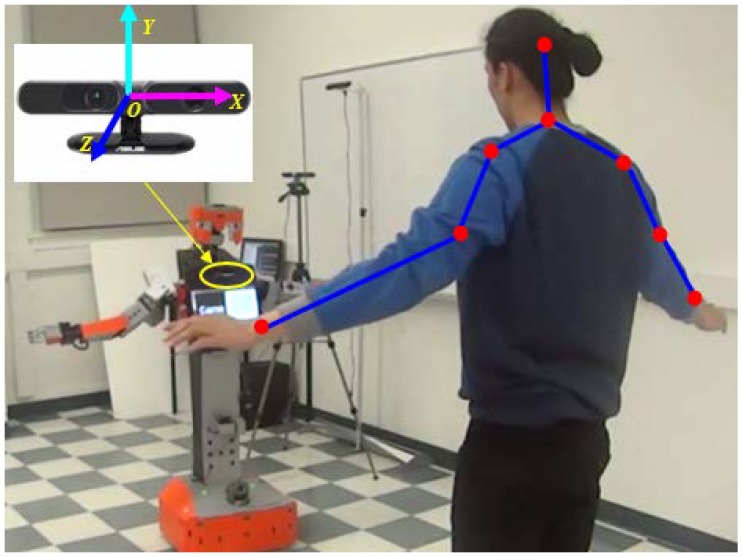
The Tangy robot using the ASUS Xtion 3D sensor to learn human demonstrator motions.

We implemented our gesture imitation learning system on the humanoid robot, Tangy, shown in Figure 8. The robot consists of two six-DOF arms, an upper torso with a tablet screen, a lower torso connected to a mobile base. The configuration and parameters of the robot are detailed in Appendix A. Following the bounding-volume-based approach presented in Section 2.2.1, the robot’s upper torso is modeled as five capsules (see Figure 8a), with one capsule located at the bottom of the screen and two spheres located at the two bottom corners of the screen to incorporate the USB cables, the circles shown in Figure 8b. The lower torso, the upper arms, and the forearms are also modeled as capsules, and the head is modeled as a sphere, as shown in Figure 8a. We used the ASUS Xtion 3D sensor, Figure 9, to track the position of the demonstrator’s joints using the OpenNI skeleton tracker [21]. In our real-world implementation of the gesture imitation learning system, we include angular velocity joint constraints to filter noisy sensor data which does not correspond to actual human demonstrator joint movements. Namely, an empirically identified threshold of 1 rad/s was used with our gesture imitation learning system. We have also included speed constraints to prevent the robot manipulator from exceeding 250 mm/s to comply with the current ISO 15066 for collaborative robots to ensure safety when a robot is working in close proximity to a human [22].

## 4. Experimental Section

We evaluate the performance of our proposed gesture imitation learning system both in simulation and in the real-world on the Tangy robot. Namely, the experiments focused on evaluating the imitation learning system to investigate the performance of the system in: (1) avoiding self-collisions during a human demonstration; and (2) generating collision-free robot arm trajectories that are similar to the demonstrator’s motions. 

**Figure 10:**
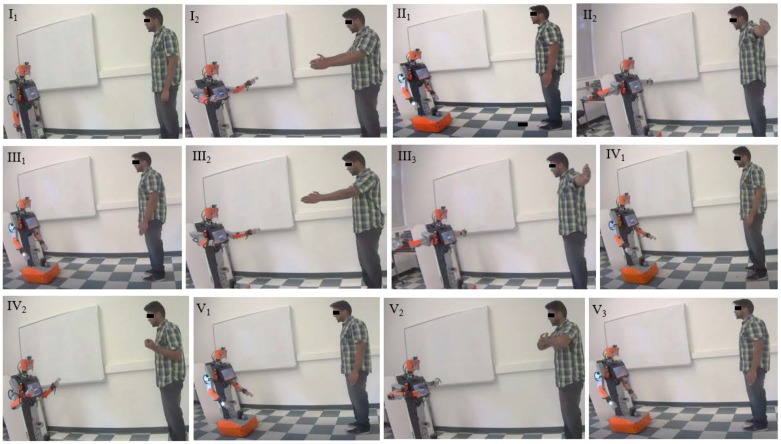
Video frame sequences when imitating human demonstrator motion using the imitation learning system. Five sets of motions include motion I (**I_1_**–**II_1_**), motion II (**II_1_**–**III_1_**), motion III (**III_1_**–**IV_1_**), motion IV (**IV_1_**–**V_1_**), and motion V (**V_1_**–**V_3_**).

Human demonstrators presented five separate motions to the robot, motions I to V, Figure 10. Motion I has the demonstrator extend his/her arms forward horizontally from the vertical position (from pose I_1_ to pose I_2_) and then back to the vertical position (from pose I_2_ to pose II_1_). motion II has the demonstrator extend his/her arms horizontally sideways from the vertical position (from pose II_1_ to pose II_2_) and then back to the vertical position (from pose II_2_ to pose III_1_). Motion III has the demonstrator extend his/her arms forward horizontally from the vertical position (from pose III_1_ to pose III_2_), then sideways (from pose III_2_ to pose III_3_), and finally back to the vertical position (from pose III_3_ to pose IV_1_). Motion IV has the demonstrator fold his/her elbow (from pose IV_1_ to pose IV_2_) and then back to the vertical position (from pose IV_2_ to pose V_1_). Motion V has the demonstrator fold his/her arms towards the chest (from pose V_1_ to pose V_2_) and then back to the vertical position (from pose V_2_ to pose V_3_). These motions were selected as they evaluate the entire range of motion for the robot’s shoulder and elbow joints. Similar motions were also evaluated in [18]. Demonstrators were asked to repeat each motion twice. 

### 4.1. Simulation Results

**Figure 11:**
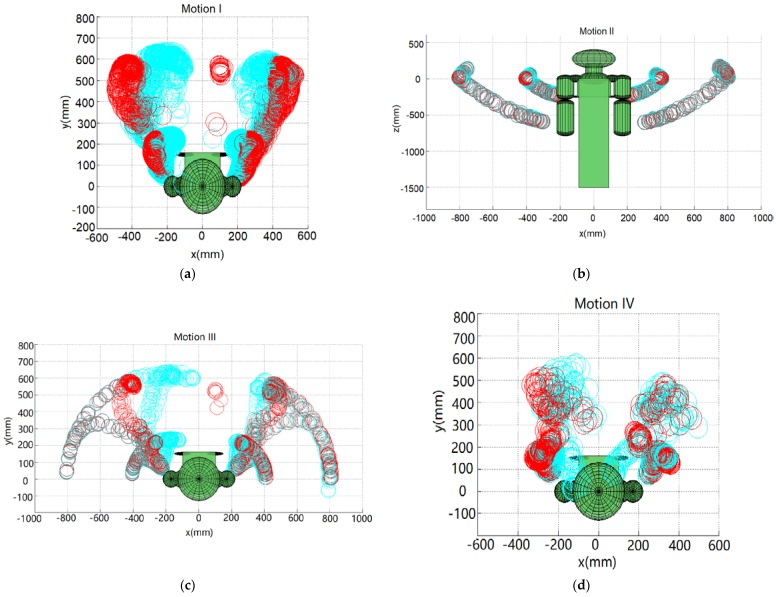

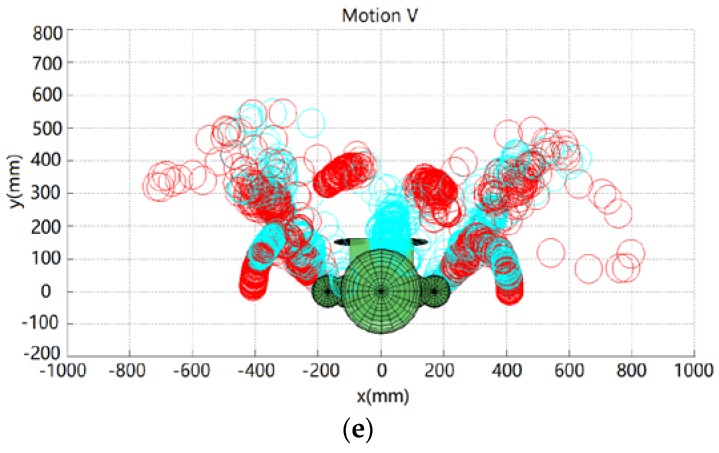
The positions of the robot’s arms for Motions I, II, III, IV and V shown in (**a**–**e**), respectively. Blue circles and red circles represent the positions determined by the directly mapped and collision-free trajectories, respectively.

The demonstrator’s five set of motions are captured using the ASUS Xtion 3D sensor. Following the geometric-based approach presented in Section 2.1, the demonstrator’s poses are mapped to the robot. Figure 11 shows the position of the robot’s end-effector and the elbow for the aforementioned five set of motions. The blue circles represent the workspace of the robot’s end-effector and elbow when the joint angles of the human demonstrator are directly mapped to the robot, ***q****_mimic_*, without any self-collision avoidance. The red circles represent the robot’s collision-free poses determined with self-collision avoidance, ***q****_collision-free_*. It can be observed from Figure 11 that some of the blue circles collide with the robot, while no red circles collide with the robot. It is noted that there is collision if the upper arm (the segment of shoulder and elbow) or the forearm (the segment of elbow and wrist) contacts any of the robot body parts. For example, without self-collision avoidance, the robot arms collide with the tablet screen in motions I, III, IV and V, and the two wrists collide with each other in motion V.

**Figure 12:**
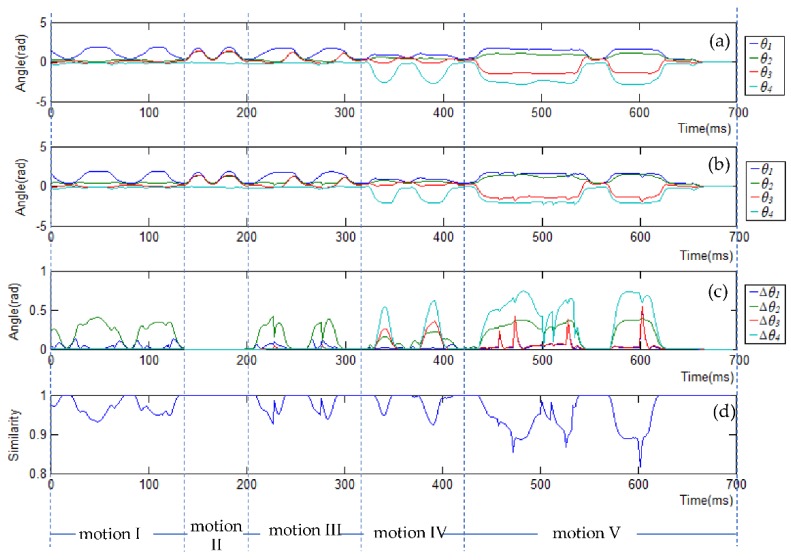
The trajectories of the left arm joint: (**a**) the trajectories of the four angles of the arm without self-collision avoidance; (**b**) collision-free trajectories of the arm; (**c**) the absolute angle difference between the trajectories in (**a**,**b**); (**d**) the similarity between (**a**) and (**b**).

Figure 12a,b show the ***q****_mimic_* and ***q****_collision-free_* trajectories for each motion, respectively. It is observed that motion I (extending arms forward) is mainly actuated by the shoulder roll angle *θ*_1_; motions II (extending arms horizontally) and III (extending arms forward and then horizontally) are actuated by all the shoulder joints, *θ*_1_, *θ*_2_, and *θ*_3_. Different from motions I, II, and III, motion IV (folding elbows) is mainly actuated by the elbow joint *θ*_4_. Motion V (a random movement) is actuated by all the joints. Figure 12c shows which joints are actuated to avoid the potential collisions. Δ is used to represent the difference between ***q****_mimic_* and ***q****_collision-free_*, i.e., Δ = **|*q****_mimic_* − ***q****_collision-free_***|**. For example, for motion I and motion III, the robot arm collide with the side of the robot’s tablet screen without self-collision avoidance, thus the collision-free trajectory stretches the arms out to move the arms away from the side of the screen (Figure 11a,c), which is achieved by changing the shoulder pitch angle *θ*_2_, as can be denoted by Δ*θ*_2_ for motion I and III in Figure 12c. For motions IV and V, there are collisions with the screen surface without self-collision avoidance, and furthermore, in motion V between the two wrists, Figure 11d,e. To avoid these collisions, the collision-free trajectory lifts up the robot arms (via changing the shoulder yaw angle *θ*_3_), straightens the arms (via changing the elbow roll angle *θ*_4_), and stretches the arms out (via changing the shoulder pitch angle *θ*_2_), as can be seen by Δ*θ*_3_, Δ*θ*_4_ and Δ*θ*_2_ for motions IV and V in Figure 12c. As there were no self-collisions for motion II (Figure 11b), the difference between ***q****_mimic_* and ***q****_collision-free_* was 0, as can be seen in Figure 12c. 

Figure 12d shows the similarity between ***q****_mimic_* and ***q****_collision-free_*. We define similarity as follows [15]:

(11) $$\;W\left(\theta_{i},\theta_{i}^{\prime }\right)=\frac{1}{1+\sum_{i=1}^{u}{\left(\frac{\theta_{i}-\theta_{i}^{\prime }}{\theta_{i\_max}-\theta_{i\_min}}\right)}^{2}},\;\;0<W\leq 1\;\;\;$$

where *θ_i_* and $\theta_{i}^{\prime }\;$are, respectively, the demonstrated joint angle and collision-free joint angle of joint *i*, *u* is the total number of robot joints, and *θ_i_max_* and *θ_i_min_* are the maximum and the minimum angle of joint *i*. A larger *W* represents a higher similarity between ***q****_collision-free_* to ***q****_mimic._* Namely, if *W* = 1, the collision-free pose is the same as the demonstrated pose. From Figure 12d, it can be seen that the similarity for all the motions are close to 1, that is, the collision-free trajectory is close to the trajectory that is directly mimicked. 

### 4.2. Real-World Results with Tangy

The Tangy robot’s poses during human demonstration using the imitation learning system are also shown in Figure 10 for all five motions. In particular, the figure shows the robot’s arm poses during one entire demonstration session. In general, all potential collisions were avoided in real-time for all five demonstrators. 

## 5. Conclusions

In this paper, we developed a gesture imitation learning system with self-collision avoidance using a 3D sensor. The performance of our imitation learning system with self-collision avoidance was evaluated in both simulation and on the physical robot Tangy. System performance results comparing trajectories generated by the imitation learning system to directly mapping demonstrator trajectories without self-collision showed that our proposed imitation learning system was capable of avoiding all self-collisions and generated collision-free arm trajectories that were similar to the human demonstrator motions. Furthermore, the physical robot was able to mimic the arm motions of several demonstrators in real-time when using the proposed imitation learning system. 

Our current imitation learning system does not mimic the demonstrated joint angular accelerations of a gesture. As a future step, we will consider learning from the human demonstrations these accelerations because a study has shown that the level of acceleration exhibited by a robot is related to how a human interprets the affect of the robot [23]. 

## Figures and Tables

**Figure A1:**
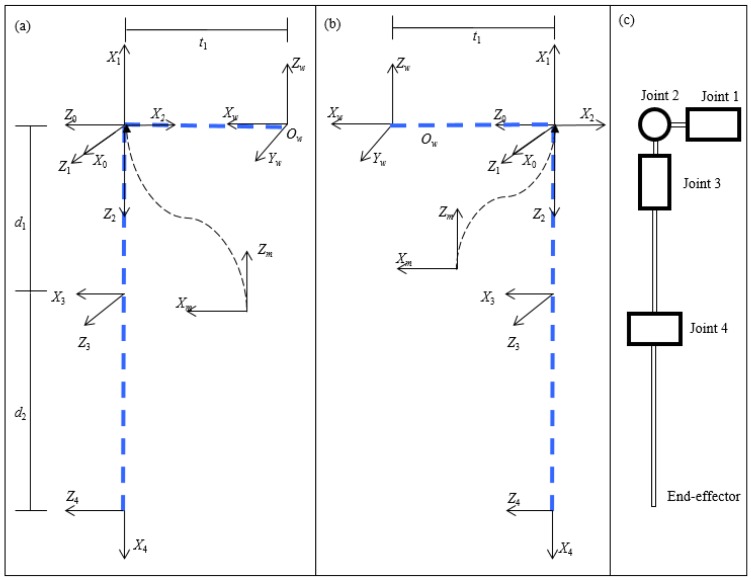
The D-H convention of the Tangy robot: (**a**) right arm; (**b**) left arm; (**c**) the joints and linkages diagram of the Tangy robot arm.

## Appendix A. Kinematics Model of the Tangy Robot Arm

The kinematic description of Tangy robot arms is modeled by assigning a coordinate system to each joint with its z-axis along the axis of rotation, following the rules of the Denavit-Hartenberg convention, as shown in Figure A1. Frame {0}, frame {1}, frame {2} and frame {3} are the frames assigned for shoulder roll, shoulder pitch, shoulder yaw and elbow roll, respectively. Frame {4} and frame {*m*} are fixed frames assigned in the end-effector and shoulder, respectively. Frame {*w*} is the world frame assigned in the center of the torso, as shown in Figure A1b.

The transformation from link coordinate frame {*i*−1} to frame {*i*} is represented by a 4 × 4 homogeneous matrix, as shown in Equation (A1), where *i* = 0, …, 4. *θ_i_*, *a_i_*, *d_i_* and *α_i_* are the joint angle, link length, offset and twist angle, respectively:

(A1) Tii−1=[cos(θi)−sin(θi)∗cos(αi)sin(θi)∗sin(αi)ai∗cos(θi)sin(θi)cos(θi)∗cos(αi)−cos(θi)∗sin(αi)ai∗sin(θi)0sin(αi)cos(αi)di0001] 

The first three elements in the last column represent the origin position of frame {*i*−1} with respect to frame {*i*}.

The Denavit-Hartenberg (D-H) parameters of the Tangy robot arms are described in Table A1 and Table A2. It is noted that *d*_1_ = 239 mm; *d*_2_ = 410 mm; *t*_1_ = 170 mm. *d*_1_ and *d*_2_ are the length of upper arm and forearm, respectively. *t*_1_ is the distance between frame {*m*} and frame {*w*}. The first two sets of D-H parameters for frame {*m*}, and frame {0} in Table A1 and Table A2 are the description of frame {*m*} in frame {*w*}, and the description of frame {0} in frame {*m*}.

The homogeneous transformation relating frame {3} (elbow frame) and frame {4} (end-effector frame) to frame {*w*} is denoted by Equations (A2) and (A3), respectively:

(A2) T3w=Tmw·T0m· T10·T21·T32 

(A3) T4w=T3w·T43

Based on the D-H parameters in Table A1 and Table A2 and the homogeneous transformations, T3w and T4w are derived. The *x*-*y*-*z* positions of the elbow and shoulder (the first three elements in the last column of T3w and T4w) are derived. The positions of the left elbow and left end-effector are represented as follows:

*x_elbow_left* = − *t*_1_ − *d*_1_ × *cos* (*θ*_2_ − π)

*y_elbow_left* = −*d*_1_ × *cos* (*θ*_1_ − π) × *cos* (*θ*_2_ − π)

*z_elbow_left* = −*d*_1_ × *cos* (*θ*_1_ − π) × *cos* (*θ*_2_ − π)

*x_end_effector_left* = *d*_2_ × *cos* (*θ*_2_ − π) × *cos* (*θ*_4_ − π) − *d*_1_ × *cos* (*θ*_2_ − π) − *t*_1_ − *d*_2_ × *cos* (*θ*_3_ − π) × *cos* (*θ*_4_ − π) × *cos* (*θ*_2_ − π)

*y_end_effector_left* = *d*_2_ × *cos* (*θ*_1_ − π) × *cos* (*θ*_2_ − π) × *cos* (*θ*_4_ − π) − *d*_2_ × *cos* (*θ*_4_ − π) × (*cos* (*θ*_1_ − π) × *cos* (*θ*_3_ − π) − *cos* (*θ*_1_ − π) × *cos* (*θ*_2_ − π) × *cos* (*θ*_3_ − π)) − *d*_1_ × *cos* (*θ*_1_ − π) × *cos* (*θ*_2_ − π)

*z_end_effector_left* = *d*_2_ × *cos* (*θ*_4_ − π) × (*cos* (*θ*_1_ − π) × *cos* (*θ*_3_ − π) + *cos* (*θ*_2_ − π) × *cos* (*θ*_3_ − π) × *cos* (*θ*_1_ − π)) − *d*_1_ × *cos* (*θ*_1_ − π) × *cos* (*θ*_2_ − π) + *d*_2_ × *cos* (*θ*_1_ − π) × *cos* (*θ*_2_ − π) × *cos* (*θ*_4_ − π)

The positions of the right elbow and the right end-effector are represented as follows:

*x_elbow_right* = *t*_1_ − *d*_1_ × *cos* (*θ*_6_ + π)

*y_elbow_right* = *d*_1_ × *cos* (*θ*_6_ + π) × *cos* (*θ*_5_ − π)

*z_elbow_right* = *d*_1_ × *cos* (*θ*_6_ + π) × *cos* (*θ*_5_ − π)

*x_end_effector_right* = *t*_1_*− d*_1_ × *cos* (*θ*_6_ + π) + *d*_2_ × *cos* (*θ*_6_ + π) × *cos* (*θ*_8_ − π) + *d*_2_ × *cos* (*θ*_6_ + π) × *cos* (*θ*_7_ − π) × *cos* (*θ*_8_ − π)

*y_end_effector_right* = *d*_1_ × *cos* (*θ*_6_ + π) × *cos* (*θ*_5_ − π) − *d*_2_ × *cos* (*θ*_8_ − π) × (*cos* (*θ*_5_ − π) × *cos* (*θ*_7_ − π) − *cos* (*θ*_6_ + π) × *cos* (*θ*_5_ − π) × *cos* (*θ*_7_ − π)) − *d*_2_ × *cos* (*θ*_6_ + π) × *cos* (*θ*_5_*−* π) × *cos* (*θ*_8_*−* π)

*z_end_effector_right* = *d*_1_ × *cos* (*θ*_6_ + π) × *cos* (*θ*_5_ − π) + *d*_2_ × *cos* (*θ*_8_ − π) × (*cos* (*θ*_5_ − π) × *cos* (*θ*_7_ − π) + *cos* (*θ*_6_ + π) × *cos* (*θ*_7_ − π) × *cos* (*θ*_5_ − π)) − *d*_2_ × *cos* (*θ*_6_ + π) × *cos* (*θ*_5_ − π) × *cos* (*θ*_8_ − π)

**Table A1:** The D-H Parameters for the Left Arm.

Frames	*θ_i_* (rad)	*d_i_* (mm)	*a_i_* (mm)	*α_i_* (rad)
Frame {*m*}	0	0	−*t*_1_	0
Frame {0}	π	0	0	π
Frame {1}	*θ*_1_ − π	0	0	π
Frame {2}	−*θ*_2_ + π	0	0	π
Frame {3}	−*θ*_3_ + π	*d*_1_	0	−π
Frame {4}	*θ*_4_ − π	0	*d*_2_	π

**Table A2:** The D-H Parameters for the Right Arm.

Frames	*θ_i_* (rad)	*d_i_* (mm)	*a_i_* (mm)	*α_i_* (rad)
Frame {*m*}	0	0	*t*_1_	0
Frame {0}	π	0	0	π
Frame {1}	*θ*_5_ − π	0	0	π
Frame {2}	*θ*_6_ + π	0	0	π
Frame {3}	−*θ*_7_ + π	*d*_1_	0	−π
Frame {4}	*θ*_8_ − π	0	*d*_2_	π

With the positions of the elbow and hand, the distance between two modeled robot body parts can be derived based on Equation (9).
